# Stimulating Multiple-Demand Cortex Enhances Vocabulary Learning

**DOI:** 10.1523/JNEUROSCI.3857-16.2017

**Published:** 2017-08-09

**Authors:** Magdalena W. Sliwinska, Inês R. Violante, Richard J.S. Wise, Robert Leech, Joseph T. Devlin, Fatemeh Geranmayeh, Adam Hampshire

**Affiliations:** ^1^Department of Medicine, Imperial College London, London, W12 0NN, United Kingdom, and; ^2^Department of Experimental Psychology, University College London, London, WC1H 0AP, United Kingdom

**Keywords:** dorsal anterior cingulate cortex, functional magnetic resonance imaging, midline superior frontal gyrus, multiple-demand cortex, novel vocabulary learning, transcranial magnetic stimulation

## Abstract

It is well established that networks within multiple-demand cortex (MDC) become active when diverse skills and behaviors are being learnt. However, their causal role in learning remains to be established. In the present study, we first performed functional magnetic resonance imaging on healthy female and male human participants to confirm that MDC was most active in the initial stages of learning a novel vocabulary, consisting of pronounceable nonwords (pseudowords), each associated with a picture of a real object. We then examined, in healthy female and male human participants, whether repetitive transcranial magnetic stimulation of a frontal midline node of the cingulo-opercular MDC affected learning rates specifically during the initial stages of learning. We report that stimulation of this node, but not a control brain region, substantially improved both accuracy and response times during the earliest stage of learning pseudoword–object associations. This stimulation had no effect on the processing of established vocabulary, tested by the accuracy and response times when participants decided whether a real word was accurately paired with a picture of an object. These results provide evidence that noninvasive stimulation to MDC nodes can enhance learning rates, thereby demonstrating their causal role in the learning process. We propose that this causal role makes MDC candidate target for experimental therapeutics; for example, in stroke patients with aphasia attempting to reacquire a vocabulary.

**SIGNIFICANCE STATEMENT** Learning a task involves the brain system within which that specific task becomes established. Therefore, successfully learning a new vocabulary establishes the novel words in the language system. However, there is evidence that in the early stages of learning, networks within multiple-demand cortex (MDC), which control higher cognitive functions, such as working memory, attention, and monitoring of performance, become active. This activity declines once the task is learnt. The present study demonstrated that a node within MDC, located in midline frontal cortex, becomes active during the early stage of learning a novel vocabulary. Importantly, noninvasive brain stimulation of this node improved performance during this stage of learning. This observation demonstrated that MDC activity is important for learning.

## Introduction

Learning mechanisms in the human brain involve an interplay between qualitatively distinct large-scale networks, both domain-specific and domain-general (Jueptner et al., 1997; Honda et al., 1998; Köhler et al., 1998; Petersson et al., 1999; Chein and Schneider, 2005, 2012; Duncan, 2010). Domain-specific networks encompass brain regions that are highly specialized for particular types of demand, for example, motor or language processes (Varley et al., 2005; Fedorenko et al., 2011; Monti et al., 2012). By contrast, domain-general networks encompass brain regions that are involved in processes that apply to a wide variety of contexts, including those that underlie working-memory, reasoning, attention, and executive function. The most commonly activated domain-general networks collectively form multiple-demand cortex (MDC). MDC is particularly active during novel or complex tasks, which cannot be performed automatically (Duncan and Owen, 2000; Fedorenko et al., 2013).

There has been increasing interest in understanding the role of MDC as part of the neural mechanism that supports the human capacity for rapid learning. Neuroimaging studies have reported strong activation in MDC during the learning of word lists (Andreasen et al., 1995; Kopelman et al., 1998; Wildgruber et al., 1999), object-location associations (Büchel et al., 1999), noun–verb associations (Raichle et al., 1994), abstract designs (Petersson et al., 1999), abstract shapes (Chein and Schneider, 2005), faces (Wiser et al., 2000), sequential finger movements (Jenkins et al., 1994; Doyon et al., 1996), and arbitrary stimulus–response rules (Toni et al., 2001; Hampshire et al., 2016). These diverse studies have shown that a characteristic of the response of MDC are reliable reductions in the extent and magnitude of within-networks functional activity and connectivity between the early and late stages of learning (Toni et al., 2001; Hampshire et al., 2016).

It has been proposed that these are the interactions between domain-specific and MDC that enable rapid and successful learning (Chein and Schneider, 2005, 2012). They may be understood as a trainer–operator synergy. When initially faced with a learning task, MDC is recruited and establishes a temporary program for performing the task. The rapid formation of that program in and of itself is a complex multistage process, involving fine tuning through prediction and outcome monitoring (Ruge and Wolfensteller, 2016). Once formed, this program enables the task to be performed with high accuracy after learning, whether from trial-and-error or error-free after simple instruction. Simultaneously, it provides a top-down template that accelerates longer-term learning and eventual automatization of the task within domain-specific networks. A logical prediction of this hypothesis is that interventions that target MDC should modulate the rate and accuracy of learning at the earliest stages, potentially across a wide variety of behavioral domains.

Here we report findings from two studies that used a combination of neuroimaging and transcranial neurostimulation to test this prediction in the context of novel vocabulary learning. In the first study, functional magnetic resonance imaging (fMRI) was used to confirm whether a target MDC network was selectively activated in the early stages of vocabulary learning. This network comprised midline superior frontal gyrus and adjacent dorsal anterior cingulate cortex (SFG/dACC), and bilateral anterior insular brain regions. In the second study, transcranial magnetic stimulation (TMS) was applied to either the SFG/dACC node or to a control site that had been observed to deactivate during the learning task in the fMRI Study. The impact of stimulation on either site on performance of the vocabulary learning was measured. This determined whether the functionality of the MDC node was causally linked to performance speed or accuracy during different stages of the learning process.

## Materials and Methods

### 

#### Participants

Twenty native English speakers (11 women, aged 21–65 years; average, 31) participated in the fMRI Study. Of these 11 (7 women, aged 22–65 years; average, 33) participated in the subsequent TMS Study together with four additional native English speakers (2 women, aged 25–46 years; average, 32). The stimulus sets differed across studies and there was an inter-study gap of at least 11 weeks for those participants who took part in both studies. The behavioral data collected from one participant in the fMRI Study were lost due to a technical failure. All volunteers had normal or corrected-to-normal vision and normal hearing. They reported having no neurological or language impairments. Each person gave written informed consent before participation in this study. Ethical approval for the fMRI Study was granted by NRES Committee London-West London & GTAC and for the TMS Study by the University College London Research Ethics Committee.

#### fMRI Study: experimental design and statistical analyses

##### Experimental procedure.

Each participant attended one fMRI session which lasted ∼23 min. During this session, participants performed two experimental tasks. These were as follows: (1) a main novel vocabulary learning task, and (2) a control real word–object matching task. A short practice version of the experimental tasks was performed in the scanner before testing. This was done to ensure that participants were familiar with the tasks and that auditory stimuli were clearly heard.

##### Novel vocabulary learning task.

During the novel vocabulary learning task, participants were required to learn associations linking colored pictures of objects to pseudowords, comprising normal English phonology (e.g., a picture of a tree, *flen*, or a picture of a frog, *tync*). Without prior training in the novel vocabulary, participants were asked to judge whether a novel word they heard was the “correct” name for an object that they saw on a screen. After each trial, participants received written feedback on their performance, which informed them whether their answer was correct or incorrect, and regardless of accuracy it provided them with the correct name of the object. If participants did not respond on time, the feedback informed them about missing a trial and still provided them with the correct name of the object. Examples of feedback slides are as follows: *Correct! This is flen*; or *Incorrect! This is flen*; or *Missed! This is flen*). The feedback trained participants in the pseudoword–object associations on each trial and over repeated trials, using both instruction-based and reinforcement-based mechanisms. When the object was shown for the first time, participants were told to guess whether the association with the heard pseudoword was correct or incorrect, and use the feedback to gain information about the new name for this object.

**Figure 1.**
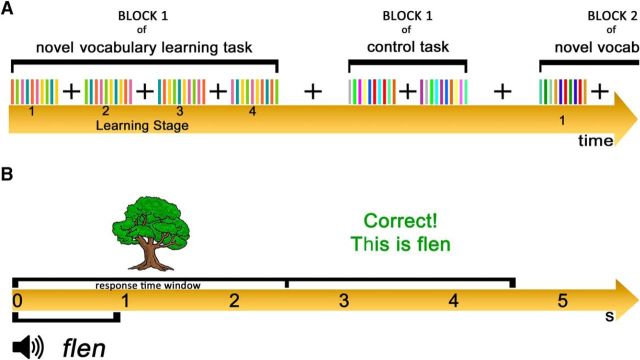
Experimental design used in the fMRI Study. ***A***, The stimuli were presented using an alternating block design and each task had four blocks. In the novel vocabulary learning task, stimuli within each block were organized into four learning stages of equal length. ***B***, Each trial lasted 4.5 s, starting with presentation of the picture of the object for 2.5 s and simultaneous auditory presentation of a pseudoword or real word, depending on the task, for ∼1 s. Within the first 2.5 s, participants were also required to respond to the stimulus. After the first 2.5 s visually presented feedback was displayed for the remaining 2 s.

In total, participants learned four different stimulus sets, each of which consisted of five pseudoword–object associations. Each set was learnt in a 3 min block of 40 trials (Fig. 1*A*). This sequential design dissociated learning effects from other potentially confounding factors related to time on task, novelty of being in the scanner and fatigue. In each block, the 40 trials were organized into four learning stages (Learning Stage 1–4) of equal length separated by presentation of a fixation cross. Within each learning stage, each object was presented pseudorandomly two times, once with the correct and once with the incorrect association. During each learning block there were a list of five correct pseudoword–object associations and a different list of 10 pseudowords forming incorrect association trials. The latter were each presented twice across the learning block, pseudorandomly paired with two objects. The same pictures of an object were never presented twice in a row.

Each trial lasted for 4.5 s (Fig. 1*B*). Trials started with a simultaneous presentation of the object and the pseudoword. The object was presented for 2.5 s and the heard pseudoword for ∼1 s. The object presentation was followed by a feedback slide which was displayed on the screen for the remaining 2 s. Therefore, participants had to provide their response within the first 2.5 s. They were advised to respond as quickly and accurately as possible by pressing appropriate buttons on the response box using their left and right index fingers. Rest trials during which participants were asked to look at a fixation cross were included as a low-level baseline condition. A single rest trial lasted for the duration of an experimental trial (4.5 s), and was presented after every 10 experimental trials within each learning block. After each learning block, a rest trial was presented for 18 s to allow switching to the control task. In addition, at the beginning of each block there was a short written instruction reminding participants about the task that was to be performed.

To ensure that participants made every effort to learn new associations, a vocabulary test was applied at the end of scanning, and participants were informed that this would happen before scanning commenced. During this delayed test, participants were shown pictures of objects that were used in the learning task and were asked to provide a newly learnt name for each object without any cues. Because the test was only used to motivate participants to learn, and the time from task to test could vary, the results were not analyzed.

##### Control real word–object matching task.

During the control real word–object matching task, participants were asked to determine whether a heard English word was correctly paired with the picture of an object. This task was designed to control for the stimulus processing, motor, and “yes/no” decision processes of the main task, but in the absence of new vocabulary learning. In accordance with the main task, feedback was provided at the end of each trial. Participants performed four blocks of this task with four different stimulus sets (Fig. 1*A*). Each block consisted of only 20 trials, as it was known from a pilot study that there were no learning effects on this task. Therefore, there was no need for a greater number of trials to observe learning over time. The objects in the control task were presented pseudorandomly two times within each block, once with the correct and once with the incorrect real word–object association. Each block had a list of 10 correct real word–object associations and 10 real word–object pairings that formed incorrect association trials, and these were pseudorandomly paired with only one picture within a block. Timing parameters for the control task were matched to those of the main task.

##### Stimuli.

Three types of stimuli were used: pictures of objects, auditory recordings of pseudowords, and auditory recordings of real English words. Pictures were of well known objects that represented monosyllabic nouns (e.g., tree, frog, bed). They were selected from a standardized picture set (Snodgrass and Vanderwart, 1980; Rossion and Pourtois, 2004). Pseudowords were monosyllabic and respected the orthographic and phonotactic rules of English (e.g., flen, tync, deik). They were generated using the ARC Nonword Database (Rastle et al., 2002). Real words were monosyllabic names of well known objects. All real English words, including names of visually presented objects, were matched across the scanning blocks (fMRI Study) and stimulation runs (TMS Study) with respect to imageability, concreteness, familiarity, and Kucera–Francis frequency based on measures taken from the Medical Research Council Psycholinguistic database (Wilson, 1988).

##### Stimuli presentation.

The paradigm was programmed using MATLAB Psychophysics Toolbox (Psychtoolbox-3; www.psychtoolbox.org). The visual stimuli were presented in black 80 point bold Arial font via IFIS-SA system (In Vivo). The feedback was presented using the same size and theme of the font but in different colors: green was used for correct responses; red for incorrect responses; and dark blue for missed trials. The auditory stimuli were presented using Sensimetrics S14 sound-attenuating in-ear MR-compatible headphones. Responses were recorded through a fiber optic response box (NordicNeuroLab), interfaced with the stimulus presentation PC running MATLAB.

##### Image acquisition.

Whole-brain imaging was performed on a Siemens 3.0 Tesla Verio MR scanner. A high-resolution T1-weighted anatomical scan (160 interleaved slices; voxel size: 1.0 × 1.0 × 1.0 mm; TR: 2300 ms; TE: 2.98 ms; TI: 900 ms; flip angle: 9°; field of view: 256 × 240 × 160 mm) was obtained for each participant. The functional data were acquired using T2-weighted gradient-echo EPI sequence (35 interleaved slices; voxel size: 3.0 × 3.0 × 3.0 mm; TR: 4500 ms; TE: 30 ms; flip angle: 80°; delay in TR: 2500 ms; field of view: 192 × 192 × 105 mm). To reduce auditory interference from the noise of the scanner with the auditory presentation of words, a semisparse sampling design (Leech et al., 2009) was used in each trial. This meant that the volume acquisition began 2.5 s after stimulus onset and lasted for another 2 s. In the whole run, 296 volumes were acquired.

##### Behavioral data analyses.

Both accuracy and reaction time (RT) data were analyzed individually for each task to confirm that (1) participants were learning the pseudoword–object associations across four learning stages of each block of the novel vocabulary learning task, (2) there was no performance improvement within sets of the control task, and (3) there was no performance improvement due to increasing familiarity with the tasks over the four stimulus sets (i.e., meta-learning). In the novel vocabulary learning task, mean accuracy and median RT for each participant per learning stage were analyzed at the group level using a 4 × 4 repeated-measures ANOVA with Stimulus Set (1–4) and Learning Stage (1–4) as independent factors. In the real word–object matching task, trials within each set were divided into four consecutive stages and group analyses of mean accuracy and median RT for each participant per task stage were analyzed at the group level using a 4 × 4 repeated-measures ANOVA with Stimulus Set (1–4) and Task Stage (1–4) as independent factors. *Post hoc* paired two-tailed *t* tests (with Bonferroni correction for multiple comparisons) were used to characterize the significant findings. Only median RT for correct responses was used in the statistical analyses to minimize the effect of outliers. The initial guess trials for the first learning stage were excluded as these were at chance, as expected.

##### Neuroimaging data analyses.

Two sets of analyses were performed. The aim of the first analysis was to identify brain regions with activation that was greater for the learning than the control task. To achieve this, the brain activation across all learning trials was contrasted with activation across all trials of the control task. The aim of the second analysis was to identify brain regions that demonstrated significant differences in blood oxygen level-dependent (BOLD) activation across four learning stages during the novel vocabulary learning task. To achieve this, the brain activation during each learning stage was analyzed using a one-way repeated-measures ANOVA with Learning Stage (1–4) as a main factor. To examine Learning Stage effect further, region-of-interest (ROI) analyses were performed. The mean BOLD signal during each learning stage and the control task was extracted for major clusters defined by the ANOVA results in every participant. It was then compared between learning stages for each ROI using paired two-tailed *t* tests (with Bonferroni correction for multiple comparisons).

All functional data were analyzed using fMRI Expert Analysis Tool included in FMRIB (v6.0) Software Library (www.fmrib.ox.ac.uk/fsl). As part of the prestatistical processing, single-subject functional images underwent extraction of nonbrain structures performed with Brain Extraction Tool (BET). In addition, interleaved slice timing correction, MCFLIRT motion correction, spatial smoothing with a 5 mm full-width half-maximum Gaussian kernel, high-pass temporal filtering, and pre-whitening were applied to the data. In the first-level analysis, time-series analyses were performed on the preprocessed functional images using the general linear model to compute subject-specific patterns of activation. In the first set of analyses, the independent predictors of Learning Task, Control Task, and Instructions were entered into the model, as well as six movement regressors to account for movement-related noise. The model was convolved using double-gamma hemodynamic response function (HRF) and temporal derivatives for each predictor, with the exception of motion regressors, were included. Because the main aim of these analyses was to identify brain regions with significant activation specific to the learning task, the linear contrast of (Learning Task > Control Task) was computed. In the second set of analyses, four predictors, one for each stage of the learning task, were entered into the model. Similarly, this model was convolved to include double-gamma HRF and temporal derivatives for each predictor. Correct, incorrect, and missed trials were included in the analyses because participants were able to learn novel associations on each trial based on feedback. For the ROI analyses, regional masks were created using a sphere with 5 mm radius centered at the peak coordinates provided by the ANOVA analysis.

Functional images for each participant were registered to their anatomical scan and then to the Montreal Neurological Institute (MNI) 152-mean brain using a 12 degree-of-freedom affine registration. After the first-level analyses were completed, individual subject results were entered into higher-level group analyses where(Learning Task > Control Task) contrast and a one-way repeated-measures ANOVA were performed using a mixed-effects model (with FLAME 1). All analyses were conducted at the whole-brain level. Differences between conditions were considered significant at *Z* > 2.3 and cluster *p* < 0.05, using a clusterwise significance test.

#### TMS Study: experimental design and statistical analyses

##### Experimental procedure.

**Figure 2.**
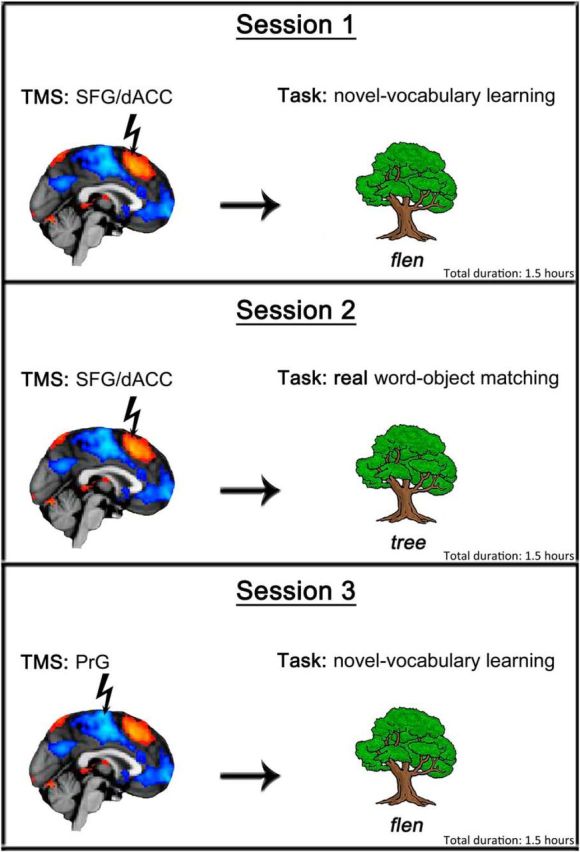
The three TMS sessions performed by each participant. Session 1 involved TMS to SFG/dACC site and the novel vocabulary learning task. Session 2 involved TMS to SFG/dACC site and the control real word–object matching task. Session 3 involved TMS to the control PrG site and the novel vocabulary learning task.

Participants received TMS on three occasions (Sessions 1–3; Fig. 2), with each occasion separated by at least 1 week. Each session lasted ∼1.5 h. During each session, TMS was delivered off-line (when participants were not performing the task) for 10 min at a frequency of 1 Hz, with intensity set to 55% of the maximum stimulator output. In the first two sessions, the TMS was applied to the midline SFG/dACC, and the participants performed the novel vocabulary learning task (at Session 1) or the real word–object matching control task (at Session 2). Finally, during Session 3, the vocabulary learning task was performed as in Session 1, but stimulation was applied to the control site in the midline precentral gyrus (PrG). The designated task at each session was performed twice. All participants performed the task immediately after TMS (experimental run), and either immediately before TMS or 30 min after (baseline run). We expected TMS to affect task performance for ∼15–20 min after the termination of stimulation based on the previous TMS studies (Chen et al., 1997; Boroojerdi et al., 2000; Pobric et al., 2007). The order of the experimental and baseline runs within each session was counterbalanced across participants. Sessions 1, 2, and 3 were undertaken in fixed order. Each session started with a short practice run to familiarize participants with the task.

During Session 1 and 3, the participants learnt a set of 10 pseudoword–object associations over 100 trials in each run, and these trials were organized into five consecutive Learning Stages (1–5) of 20 trials each (the number of trials was greater during the TMS Study compared with the fMRI Study, to allow any later stages of learning to be determined). Ten pictures of objects within each learning stage were distributed pseudorandomly so that each object occurred twice, once with the correct and once with the incorrect association. For each run a list of 10 correct pseudoword–object associations were created. In addition, a different list of 25 pseudowords was used to form incorrect association trials, and these were presented twice each across the run, pseudorandomly paired with two objects. Stimuli lists were balanced across runs and participants. Each run lasted ∼8 min; this design ensured that the experimental runs fall well within the effective poststimulation time window.

During Session 2, the participants performed 80 trials of the control real word–object matching task in each run during which they saw 40 different pictures of objects presented twice, once paired with the correct name and once with an incorrect name. For each run, a list of 40 correct real word–object associations was created. In addition, a list of 40 different real words was used to form incorrect associations within a run, and each of these words was pseudorandomly paired with one picture. Lists of stimuli were balanced across runs and participants. Each run lasted ∼6 min, which is well within the effective poststimulation time window.

##### Transcranial magnetic stimulation.

Stimulation was performed using a Magstim Rapid2 stimulator (Magstim) and a 70 mm diameter figure-of-eight coil. The TMS frequency, intensity, and duration were well within established international safety limits (Wassermann, 1998; Rossi et al., 2009). Before a participant arrived for TMS testing stimulation targets were identified and marked on their MRI scan using the Brainsight frameless stereotaxy system (Rogue Research). During testing, a Polaris Vicra infrared camera (Northern Digital) was used in conjunction with the Brainsight frameless stereotaxy system to register the participant's head to their own MRI scan to accurately target stimulation throughout the experiment. All participants used earplugs to attenuate the sound of the coil discharge and avoid damage to the ear (Counter et al., 1991). All participants tolerated TMS well and none of them reported any uncomfortable sensations due to stimulation.

##### Behavioral data analyses.

Data in the control real word–object matching task were divided into four consecutive Task Stages (1–4) of 20 trials each, matching the learning stages of the novel vocabulary learning task. Both mean accuracy and median RT for each participant per learning/task stage were analyzed at the group level for each task to assess whether TMS had any effect on learning. The initial guess trials for the earliest learning stage were excluded.

Visual examination of the data indicated that the application of TMS had different effects on both accuracy and RT data during early stages of the experimental session relative to early stages of the control sessions. To determine whether these differences were statistically significant, the effects of stimulation calculated by subtracting data in no TMS condition from TMS condition in each stage for each participant were examined using the generalized liner model function in IBM SPSS Statistics software (v20.0), with Session (experimental or control) as an independent factor; Stage (1–5 in the experimental task and 1–4 in the control task) as a covariate; and a Session × Stage interaction. Between-subject effects were included as a nuisance variable.

To elucidate the basis of the statistically significant interactions, the accuracy and RT data were analyzed for each session separately. For Sessions 1 and 3, accuracy and RT data were analyzed in a 2 × 5 repeated-measures ANOVA, with Stimulation Condition (TMS and absence of TMS/baseline) and Learning Stage (1–5) as independent factors. For Session 2, accuracy and RT data were analyzed in a 2 × 4 repeated-measures ANOVA with Stimulation Condition (TMS and absence of TMS/baseline) and Task Stage (1–4) as independent factors. *Post hoc* paired two-tailed *t* tests (with Bonferroni correction for multiple comparisons) were used to further characterize significant main effects and interactions from the ANOVAs.

## Results

### FMRI Study

#### Behavioral data

**Figure 3.**
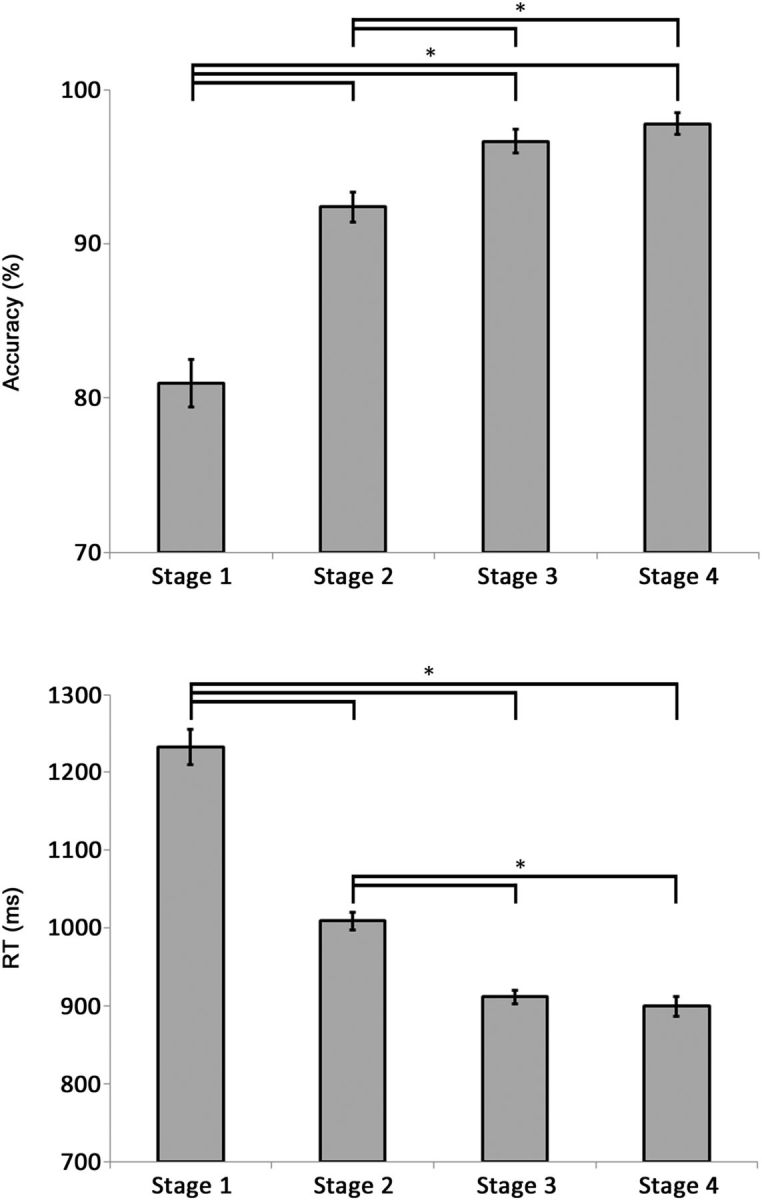
Mean group accuracy (top) and RT (bottom) during the novel vocabulary learning task performed in the fMRI Study. Error bars reflect SEM. *p < 0.01.

Overall, the results showed that in the novel vocabulary learning task there was a gradual increase of accuracy scores and a decrease of RT across the first two learning stages until these values plateaued by the third learning stage (Fig. 3). Group level analyses of accuracy data revealed a significant main effect of Learning Stage (*F*_(3,54)_ = 40.3; *p* < 0.001; partial η^2^ = 0.69), which indicated that accuracy in Learning Stage 1 (81%) was significantly lower than accuracy in Learning Stage 2 (92%), 3 (97%), or 4 (98%; all *t* tests: *t*_(18)_ > 5.24; *p* < 0.001). It also indicated that accuracy during Learning Stage 2 was significantly lower than accuracy during Learning Stages 3 or 4 (both *t* tests: *t*_(18)_ > 3.19; *p* < 0.005), but there was no significant difference in accuracy during Learning Stages 3 and 4. Group level analyses of the RT data showed a similar pattern of results. There was a significant main effect of Learning Stage (*F*_(3,54)_ = 81.32; *p* < 0.001; partial η^2^ = 0.82), with *post hoc t* tests showing responses during Learning Stage 1 (1233 ms) significantly slower than responses during Learning Stages 2 (1009 ms), 3 (912 ms), or 4 (900 ms; all *t* tests: *t*_(18)_ > 7.12; *p* < 0.001). In addition, responses during Learning Stage 2 were significantly slower than responses during Learning Stages 3 or 4 (both *t* tests: *t*_(18)_ > 6.29; *p* < 0.001), but there was no significant difference between the speed of the responses during Learning Stages 3 and 4. In addition, there were no meta-learning effects across the four sets of stimuli, as indicated by nonsignificant main effect of Stimulus Set and the two-way interaction between Stimulus Set and Learning Stage.

The control task was relatively easy to perform. Specifically, mean group results showed overall very high accuracy scores (98%) and relatively fast RT (912 ms). There was also no evidence of improved performance within the four stimuli sets, or a meta-learning effect over these stimuli sets as indicated by nonsignificant main effects of Task Stage (*F*_(54, 3)_ = 0.32, *p* = 0.81) and Stimulus Set (*F*_(54, 3)_ = 1.39, *p* = 0.26). The interaction between these two factors was also not significant (*F*_(162, 9)_ = 0.55, *p* = 0.84).

The analyses also demonstrated that participants learnt pseudoword–object associations successfully. After the second learning stage they were performing the associations with the same speed and accuracy as the real word–object associations. Comparison of group mean accuracy scores for each learning stage in the vocabulary learning task to those in the control task demonstrated that accuracies during each of the first two learning stages were significantly lower than accuracy during the control task (both *t* tests: *t*_(18)_ > 4.1; *p* < 0.001), but this difference was no longer present for the Learning Stages 3 and 4. Similarly, group mean RT during Learning Stages 1 and 2 in the learning task were significantly slower than average RT during the control task (both *t* tests: *t*_(18)_ > 4.32; *p* < 0.001), although this difference was no longer present for Learning Stages 3 and 4.

#### Neuroimaging data

**Figure 4.**
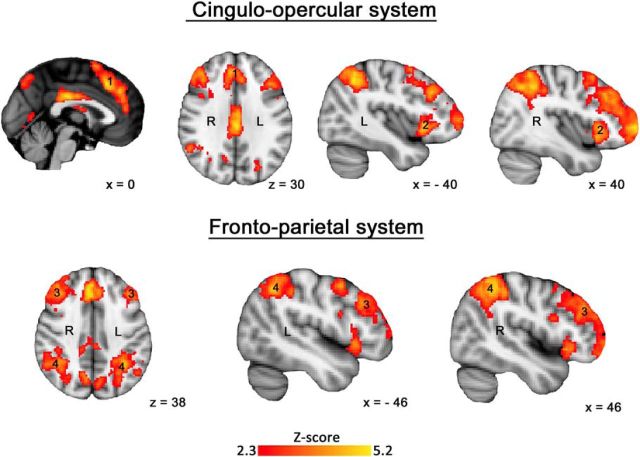
Group level *z* statistic map for the (Learning Task > Control Task) contrast overlaid on an anatomical template. Results were obtained using familywise error correction at *p* < 0.05 and cluster-forming threshold at *Z* > 2.3.

The first analysis showed significantly increased activation within the cingulo-opercular and frontoparietal networks (Fig. 4). This activation was distributed between the cingulo-opercular system, including midline SFG/dACC (Region 1) and bilateral anterior insular cortex/inferior frontal gyrus (Region 2). The frontoparietal system included bilateral dorsolateral prefrontal (Region 3), and dorsal inferior parietal (Region 4) cortices. These systems are well established as MDC (Duncan, 2013; Fedorenko et al., 2013).

**Figure 5.**
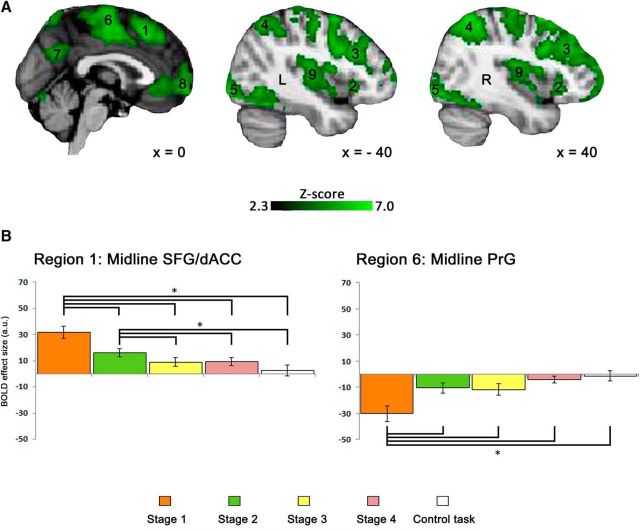
***A***, Group level *z* statistic map of all regions showing the learning stage effect overlaid on an anatomical template. Results were obtained using familywise error correction at *p* < 0.05 and cluster-forming threshold at *Z* > 2.3. ***B***, Bar plots for the BOLD signal across the four learning stages during the novel vocabulary learning task and the control task recorded for the midline SFG/dACC (left) and PrG (right). Error bars represent SEM. *p < 0.005.

The second analysis demonstrated a significant main effect of Learning Stage in cingulo-opercular and frontoparietal networks identified in the first analysis (Fig. 5*A*; Regions 1–4) as well as bilateral inferior occipital cortex (Region 5), midline PrG (Region 6), midline superior parietal cortex (Region 7), midline orbitofrontal cortex (Region 8), and bilateral posterior insular cortex (Region 9). It also confirmed that the SFG/dACC region of the cingulo-opercular system, a node of particular interest, was one of the most significantly active regions during the learning task [max *Z*-score = 5.36 at (*x*, *y*, *z*) = (0, 22, 52)].

**Figure 6.**
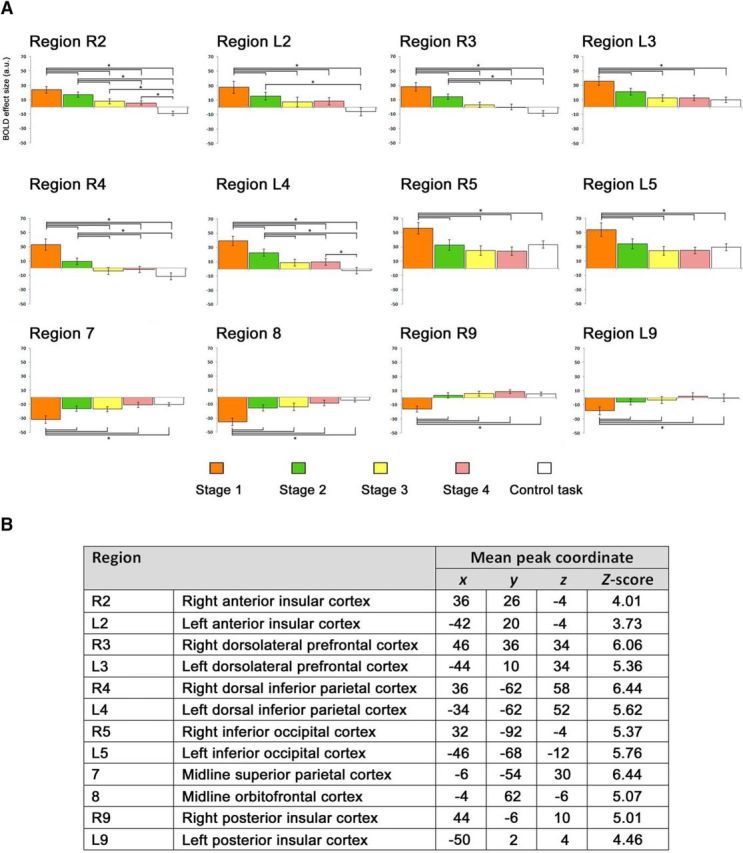
***A***, Bar plots for the BOLD signal across the four learning stages during the novel vocabulary learning task and during the control task recorded for the regions that were not presented in Figure 5. Error bars represent SEM. *p < 0.005. ***B***, The standard space (MNI) coordinates of the peak voxel and the *Z*-score for the regions presented in ***A***.

ROI analyses showed the strongest activation during the first learning stage and its gradual decrease as learning progressed in all multiple-demand regions (see Fig. 5*B* for SFG/dACC and Fig. 6*A* for the remaining regions). Specifically in SFG/dACC, the activation did not differ between Learning Stages 3 and 4, and there was no significant difference between activation in these two final learning stages and the control task. Therefore, recently learned pseudoword–object associations were no different from overlearned real word–object association as determined by either behavioral or functional imaging measures. Based on these results, we expected TMS to this site in the subsequent study to have an effect on the initial learning stages.

Figure 5*B* also presents the activation levels across the four learning stages in the region located within the midline PrG. This region was selected as a control site for the subsequent TMS Study for two reasons. First, it was deactivated throughout the entire duration of the learning task, with the most significant deactivation during the first learning stage [max *Z*-score = 5.37 at (*x*, *y*, *z*) = (0, −12, 54)]. Activation in this region gradually increased across the subsequent learning stages, but never reached zero. Therefore, we expected TMS to this site to have no effect on learning. Second, this region was closely located to the SFG/dACC site, a few centimeters posterior but still along the midline, which made it an ideal candidate for a control site as it was difficult for the participants to notice a difference in the location of stimulation.

To identify group-level MNI coordinates of the strongest activation and deactivation within SFG/dACC and PrG, respectively, we contrasted the brain activation during the first learning stage with activation during the final learning stage. Based on these results (Fig. 2), we decided to stimulate SFG/dACC at (*x*, *y*, *z*) = (−4, 12, 64) and PrG at (*x*, *y*, *z*) = (0, −24, 74) in each participant. These regions demonstrated the desired significant signal strength for the two sites while being accessible to TMS.

### TMS Study

#### Behavioral data

Analyses of TMS effects on accuracy using the generalized linear model showed a significant interaction of Session × Stage (χ^2^ = 17.12; *p* < 0.001). There was also a significant main effect of Session (χ^2^ = 29.64; *p* < 0.001) but not of Stage (χ^2^ = 3.30; *p* = 0.07). The Session × Stage interaction remained significant when including only the experimental session and control task data (χ^2^ = 6.82; *p* = 0.01), or only the experimental session and control site data (χ^2^ = 17.12; *p* < 0.001). Analyses of TMS effects on RT produced a similar result; specifically, there was a significant interaction of Session × Stage (χ^2^ = 6.62; *p* = 0.04). There was also a significant main effect of Session (χ^2^ = 10.44; *p* = 0.01) but not of Stage (χ^2^ = 1.56; *p* = 0.21). The Session × Stage interaction again remained significant when including only the experimental session and control task data (χ^2^ = 6.26; *p* = 0.01), or only the experimental session and control site data (χ^2^ = 5.29; *p* = 0.02). In addition, *post hoc t* tests showed that TMS effect on accuracy during Stage 1 of Session 1 (+ 9%) was significantly greater than TMS effect on accuracy during Stage 1 of the control Session 3 (−3%; *t*_(14)_ = 2.24, *p* = 0.03), whereas TMS effect on RT during Stage 1 of Session 1 (−105 ms) was significantly greater than TMS effect on RT during Stage 1 of the control Session 2 (+28 ms; *t*_(14)_ = 2.53, *p* = 0.02). Therefore, the effects of TMS stimulation on learning differed significantly across the experimental and control conditions.

**Figure 7.**
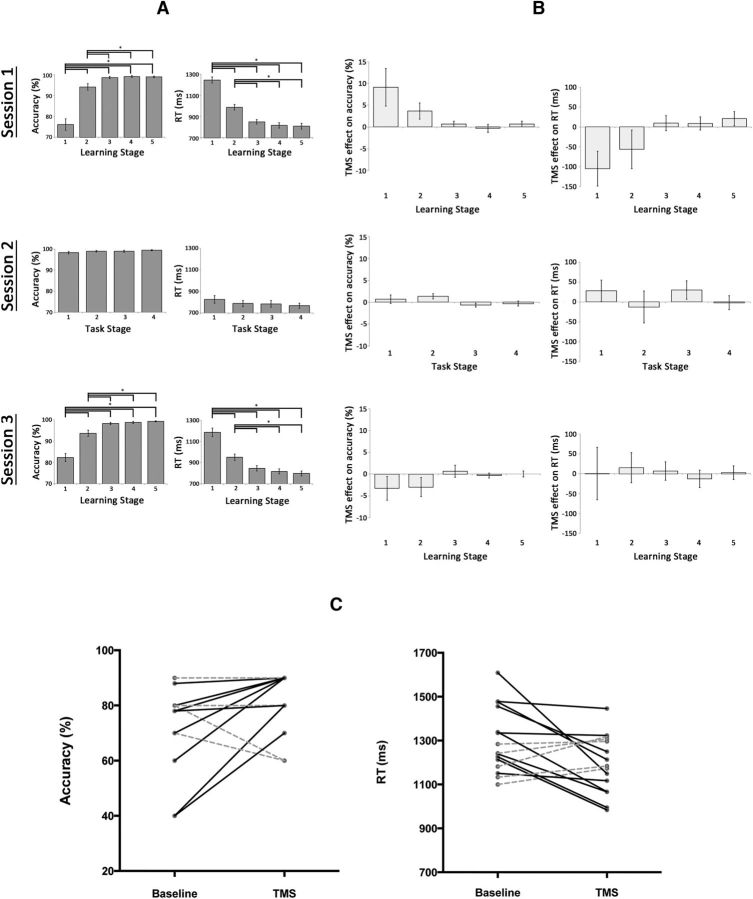
***A***, Mean group accuracy (left) and RT (right) during the novel vocabulary learning task in TMS Sessions 1 and 3, and the control real word–object matching task in Session 2. Error bars reflect SEM. *p < 0.02. ***B***, TMS effects on accuracy (left) and RT (right) represented as the difference between TMS and no TMS trials per learning/task stage for each session. Error bars represent SEM of the stimulation effect across individuals. ***C***, Individual stimulation effects during the first learning stage in Session 1. Continuous black lines indicate improved performance, whereas dashed gray lines unchanged or worsen performance.

When examining data separately for each session, for Session 1 participants demonstrated learning effects for the novel associations (Fig. 7*A*), thereby replicating the results of the fMRI Study. Analyses of accuracy showed the expected main effect of Learning Stage (*F*_(4,56)_ = 54.03; *p* < 0.001; partial η^2^ = 0.79), with *post hoc t* tests showing that accuracy during Learning Stage 1 (76%) was significantly less than during Learning Stage 2 (94%), 3 (99%), 4 (99%), or 5 (99%; all *t* tests: *t*_(14)_ > 7.89; *p* < 0.001). Similarly, accuracy during Learning Stage 2 was significantly less than during Learning Stages 3–5 (all *t* tests: *t*_(14)_ > 2.7; *p* < 0.02), whereas accuracy between Learning Stages 3–5 did not differ. Analyses of RT during Session 1 also showed the expected significant main effect of Learning Stage (*F*_(4,56)_ = 148.33; *p* < 0.001; partial η^2^ = 0.91), with *post hoc t* tests showing that RT during Learning Stage 1 (1245 ms) were significantly greater than during Learning Stages 2 (988 ms), 3 (850 ms), 4 (816 ms), or 5 (810 ms; all *t* tests: *t*_(14)_ > 10.12; *p* < 0.001). Similarly, RT during Learning Stage 2 were significantly less than during Learning Stages 3–5 (all *t* tests: *t*_(14)_ > 7.22; *p* < 0.001), whereas accuracy between Learning Stages 3–5 did not differ.

Analyses of both accuracy and RT demonstrated that TMS of the SFG/dACC had a significant enhancing effect on learning, especially during Learning Stage 1, when most learning occurred and when we expected to see the effects based on the fMRI results. First, there was a main effect of Stimulation Condition (*F*_(1,14)_ = 5.77; *p* = 0.03; partial η^2^ = 0.29) on accuracy, with greater accuracy for the TMS (95% averaged across all learning stages) than the non-TMS condition (92%). This effect was not present for RT (*F*_(1,14)_ = 1.55; *p* = 0.23; partial η^2^ = 0.1), but more critically there was a significant interaction between Stimulation Condition and Learning Stage in the accuracy (*F*_(4,56)_ = 3.38; *p* = 0.02; partial η^2^ = 0.19) and RT (*F*_(4,56)_ = 3.62; *p* = 0.01; partial η^2^ = 0.21), indicating that TMS affected learning during selected learning stages. *Post hoc t* tests showed that accuracy in Learning Stage 1 was significantly greater under the influence of TMS (81%) than without TMS (72%; *t*_(14)_ = 2.1; *p* = 0.05; Cohen's effect size *d* = 0.54). This facilitatory effect of 9% was represented as the difference in accuracy between TMS and no TMS trials per learning stage (Fig. 7*B*, left, top) and was present in 10 (of 15) participants (Fig. 7*C*, left). In Stage 2, accuracy under the influence of TMS (96%) was numerically greater than no TMS (92%), but the difference did not reach statistical significance. Accuracy during Learning Stages 3, 4, and 5 was numerically similar for the TMS and non-TMS conditions. *Post hoc t* tests also showed that responses during Learning Stage 1 were significantly faster under the influence of TMS (1193 ms) compared with the responses in the absence of an influence of TMS (1298 ms; *t*_(14)_ = 2.4; *p* = 0.03; Cohen's effect size *d* = 0.65). This facilitatory effect of 105 ms was represented as the difference in RT between TMS and no TMS trials per learning stage (Fig. 7*B*, right, top) and was also present in 10 participants (Fig. 7*C*, right). Of 15 participants, eight showed improvement in both accuracy and response times. In another two participants only accuracy was improved, whereas in another two participants only response times were improved. In the remaining three participants, TMS had no observed facilitatory effects. The effect of TMS was still apparent as a numerical difference in RT during Learning Stage 2 (TMS = 960 ms, no TMS = 1016 ms), although this difference was not significant. RT during Learning Stages 3–5 were very similar for stimulation and nonstimulation conditions.

For Session 2, group analyses of the accuracy scores and RT across five subsequent Task Stages did not show any differences in performance between the TMS and baseline runs. There was no main effect of Stimulation Condition (accuracy: *F*_(1,14)_ = 1.31; *p* = 0.27; RT: *F*_(1,14)_ = 0.31; *p* = 0.59) or Task Stage (accuracy: *F*_(3,42)_ = 1.48; *p* = 0.23; RT: *F*_(3,42)_ = 0.74; *p* = 0.48) and the interaction between these two factors was also nonsignificant (accuracy: *F*_(3,42)_ = 1.55; *p* = 0.22; RT: *F*_(3,42)_ = 0.79; *p* = 0.51). This indicates that TMS had no effect on basic choice reaction, stimulus processing or motor processing elements of the task.

When stimulation was delivered to the PrG, there were the predicted main effects of Learning Stage in accuracy data (*F*_(4,56)_ = 45.82; *p* < 0.001; partial η^2^ = 0.77) and RT data (*F*_(4,56)_ = 87.76; *p* < 0.001; partial η^2^ = 0.86), indicating gradual learning. In contrast to Session 1, TMS had no significant effect on learning. Accuracy was numerically lower when the PrG was under the influence of TMS, but this effect was not statistically significant as there was no main effect of Stimulation Condition (accuracy: *F*_(1,14)_ = 3.46; *p* = 0.08; RT: *F*_(1,14)_ = 0.01; *p* = 0.92), and there was no significant two-way interaction in accuracy (*F*_(4,56)_ = 1.04; *p* = 0.39) or RT (*F*_(4,56)_ = 0.11; *p* = 0.98) data. Similarly, contrasting accuracy or RT for Stage 1 alone showed no significant differences between stimulation and nonstimulation conditions.

It is worth noting that the facilitation effect of TMS during the first learning stage in Session 1 was significant in the group level analysis but it was not present in each tested individual. One of the reviewers suggested that the efficacy of stimulation may depend on a distance between the locations of the group level peak activation that was used by us for targeting stimulation in each participant and their individual peak activation. We did not use the individual peak activation coordinates for stimulation to keep a TMS localization procedure consistent across all individuals and identify a set of coordinates that can be used in future brain stimulation studies without prior collection of individual neuroimaging data. Nonetheless, our calculations on the group of 11 participants who took part in both studies revealed that there was a significant negative correlation between the Euclidean distance and TMS effect on accuracy (one-tailed Pearson correlation: *r* = −0.7; *n* = 11; *p* = 0.01) and a positive correlation between this distance and TMS effect on RT (*r* = 0.6; *n* = 11; *p* = 0.03). Interestingly, in those participants who showed TMS effect on both accuracy and RT, the distance between their individual and the group level activation peaks was <1.5 mm. These analyses provide additional insight into the spatial precision of TMS and stress the importance of tailoring the selection of stimulation sites on an individual basis, especially in the contexts where TMS is used as a rehabilitation tool in neurological patients.

## Discussion

The results of our neuroimaging and neurostimulation studies provide converging evidence to support the hypothesis that MDC plays an important role during the initial stages of learning a new vocabulary, when there is greatest uncertainty about accuracy. Regions within cingulo-opercular and frontoparietal networks of MDC were active during performance of the pseudoword vocabulary learning task relative to the control task, in which real word–object pairings were well established. These results accord well with findings of previous neuroimaging studies that have reported activation within these networks when a diverse range of behaviors/skills are being learnt. As expected, the activity within these regions was greatest during the initial stage of the vocabulary learning task, declining as learning progressed.

**Figure 8.**
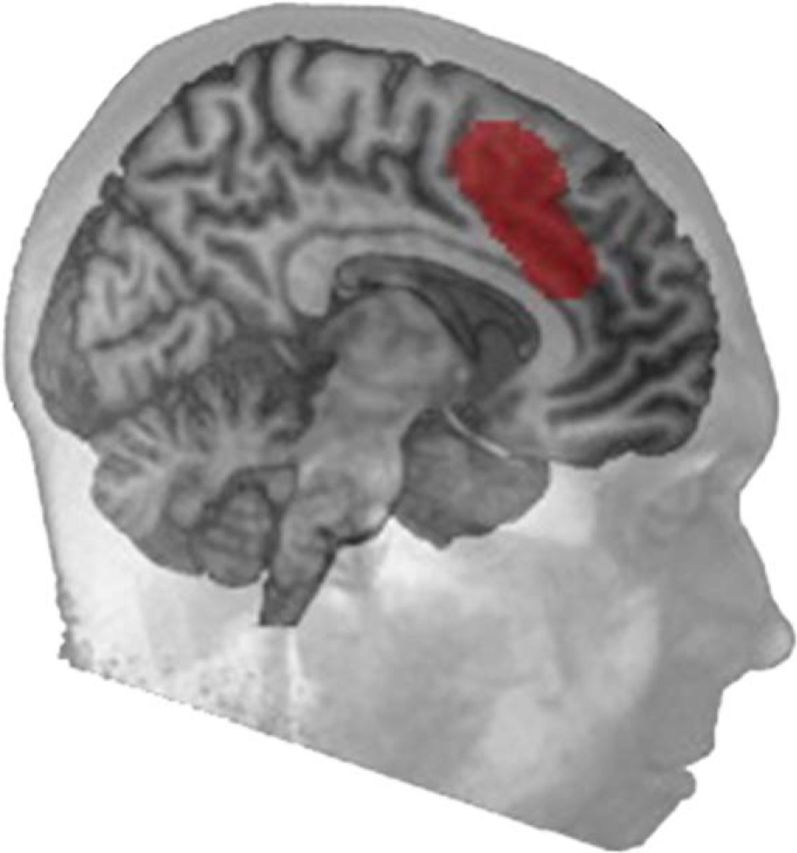
Midline frontal region of the MDC defined by Duncan (2001). Reprinted with permission from Hampshire and Sharp (2015).

Of particular relevance in the current study was the observation of learning-related activation encompassing the midline SFG/dACC and the anterior insulae. This network is active during a wide variety of cognitive tasks. It lies within the MDC, and activity within SFG/dACC recorded in our study corresponds closely to the midline frontal MDC reported in previous studies (Fig. 8; Duncan and Owen, 2000; Duncan, 2001, 2010). This region has been associated with numerous functions, including aspects of perception, response selection, executive control, working and episodic memory, and reasoning (Duncan and Owen, 2000). It is variously referred to as the cingulo-opercular (Dosenbach et al., 2008; Geranmayeh et al., 2014a) or salience network (Seeley et al., 2007; Menon and Uddin, 2010).

SFG/dACC activation gradually declined as learning progressed, and in Learning Stages 3–4 it did not differ from the control task, when pseudoword–object associations had become established and performance was no different from that on real word–object associations. This overlap between the learning curves observed in the brain relative to those observed for behavior may be accounted for by models in which MDC nodes are involved in forming a program for performing new tasks, and also in applying or monitoring that program before it becomes automated through practice (Jueptner et al., 1997; Honda et al., 1998; Köhler et al., 1998; Petersson et al., 1999; Chein and Schneider, 2005, 2012). A meta-analysis (Chein and Schneider, 2005) of the results from neuroimaging studies, variously investigating learning of many different tasks, led to the proposal that practice-related reductions in MDC activity reflected changes in the demand placed on central cognitive resources when regulating associative learning. Thus, early learning is supported and supervised by a number of interacting multiple-demand control systems that deploy, for instance, working memory, selective attention, or performance monitoring. Once learning becomes established, there is a transition from a dependence on general cognitive to more automated mechanisms.

Our TMS Study offered direct evidence for the causal role of MDC in novel vocabulary learning. Stimulation of the SFG/dACC node resulted in an acceleration of learning rate, improving both accuracy and reducing RT. No such effect was observed when targeting a control site that was deactivated during the learning task relative to the control task, providing support that SFG/dACC activity is specific to the learning process. Another study used noninvasive brain stimulation to modulate the learning of novel vocabulary in healthy participants (Flöel et al., 2008), but this study targeted the left superior temporal cortex, which is known to perform processes critical for the language domain (Geschwind, 1965; Pugh et al., 1996; Price, 2000). Our study demonstrated that stimulation of a MDC node also influences learning.

An interesting future direction would be to determine whether stimulating other MDC nodes has a similar effect on language learning. However, there are converging theoretical, practical, and clinical reasons that motivated our choice of the SFG/dACC as a target node specifically. Theoretically, there is a wealth of neuroimaging evidence to support the hypothesis that this brain region and the associated with it network are highly sensitive to learning demands (Jenkins et al., 1994; Raichle et al., 1994; Andreasen et al., 1995; Doyon et al., 1996; Kopelman et al., 1998; Büchel et al., 1999; Petersson et al., 1999; Wildgruber et al., 1999; Wiser et al., 2000; Chein and Schneider, 2005; Hampshire et al., 2016), and this was also supported by our fMRI Study. Furthermore, it has been proposed that the cingulo-opercular network may play a unique orchestrating role during learning. For example, it is particularly active when attention is switched between information coded within different posterior brain systems (Hampshire and Owen, 2006). In more direct support of its orchestrating role, analyses of directed connectivities have demonstrated that activity within this network has a causal influence on other networks. For example, it has been reported to evoke switches between MDC systems and task-independent default mode systems (Uddin, 2015). Therefore, by targeting the cingulo-opercular network we can expect to influence the broader set of networks that support the initial stages of learning (Carter et al., 1999; Chein and Schneider, 2005). On a practical level, the anatomical location of SFG/dACC within this network makes it easily accessible for direct noninvasive brain stimulation, whereas other candidate regions of the same network, such as the anterior insula, have a deeper location, which is hard to stimulate noninvasively. Last, this brain region is also of interest clinically. Studies of post-stroke aphasia have shown that the strength of activity in MDC during language processing predicts recovery from language impairments (Raboyeau et al., 2008; Brownsett et al., 2014). Indeed, given that a wealth of studies have previously reported that various aspects of language processes activate MDC (Raboyeau et al., 2008; Eckert et al., 2009; Vaden et al., 2013; Brownsett et al., 2014; Geranmayeh et al., 2014b), we believe that its cingulo-opercular network may have an important role in the recovery from various subtypes of post-stroke aphasia (Brownsett et al., 2014; Geranmayeh et al., 2014a, 2016). In fact, a parallel study from our group (Geranmayeh et al., 2017) demonstrated that in contrast to other multiple-demand areas activation specifically within SFG/dACC predicts recovery from aphasia after stroke. Therefore, the SFG/dACC may be a particularly appropriate target for therapeutic interventions. In addition, this region is usually spared by strokes resulting in aphasia, as it lies within anterior cerebral artery territory, whereas the domain-specific cortical networks for language lie within regions supplied by the middle cerebral artery.

Future research may test whether targeting the SFG/dACC modulates the balance of processing across MDC and default mode network, and whether targeting other MDC nodes has a lesser impact on learning rates. Although networks within MDC often coactivate in neuroimaging studies, they are functionally dissociable under certain conditions. Therefore, it has been proposed that they play different, albeit generalizable, functional roles (Hampshire and Owen, 2006; Dosenbach et al., 2007, 2008; Hampshire et al., 2012; Hampshire and Sharp, 2015). These networks are most active and interact in different ways in response to qualitatively distinct types of learning; for example, learning by instruction and learning through trial-and-error with feedback (Hampshire et al., 2016). We designed a task that involved both forms of learning to maximize the likelihood of modulating a learning process relevant to the task. There is some evidence (Fox et al., 2014) suggesting that stimulation of different nodes of the same network may lead to similar outcomes, but this hypothesis requires further investigation to determine whether modulating nodes of different networks impacts on different learning mechanisms.

We predict that the enhancing effects of SFG/dACC stimulation will affect other aspects of language learning, and other domains entirely, such as the learning of motor skills or abstract rules. This prediction forms the basis of further investigations, because a network with such a generalizable role would be promising as a more broadly applicable intervention target. Although this will apply to healthy volunteers, it may be extended to neurorehabilitation, based on the hypothesis that reacquisition of impaired domain-specific processes should be interpreted in terms of both domain-specific and domain-general processes.

A technical challenge for future research is to determine the optimal stimulation protocol when modulating learning. Here, we applied off-line TMS at a frequency of 1 Hz for 10 min immediately before the learning task. This eliminated nonspecific behavioral and attentional effects on the dependent measures that usually accompany on-line stimulation. However, it was unclear based on the prior literature whether to expect facilitation or retardation of learning rates. Conventional wisdom suggests that low-frequency (≤1 Hz) stimulation decreases cortical excitability, whereas high-frequency (≥1 Hz) stimulation increases excitability when applied to primary motor cortex (Pascual-Leone et al., 1994; Jennum et al., 1995; Chen et al., 1997; Berardelli et al., 1999). Outside motor cortex, studies using either high- or low-frequency repetitive TMS to areas involved in cognitive processes showed disruptive, rather than facilitatory, effects on behavior (Pascual-Leone et al., 1991; Uddin et al., 2006; Whitney et al., 2012; Sliwinska et al., 2015). In contrast, other studies demonstrated that repetitive TMS, including at the frequency used in this study protocol, can facilitate cognitive performance (Hilgetag et al., 2001; Brighina et al., 2005; Kirschen et al., 2006; Mottaghy et al., 2006; Uddén et al., 2008). A challenge for future studies will be to determine how the frequency of repetitive TMS affects task-evoked activities within the MDC.

In summary, this study demonstrated the causal role of the midline SFG/dACC in novel vocabulary learning, and the facilitatory effects on this process of low-frequency repetitive TMS applied to the SFG/dACC. This provides evidence for the importance of MDC in learning, and the possible application of this approach to neurorehabilitation in selected patients.
